# Origins of numbers: a shared language-of-thought for arithmetic and geometry?

**DOI:** 10.1016/j.tics.2025.03.001

**Published:** 2025-04-14

**Authors:** Stanislas Dehaene, Mathias Sablé-Meyer, Lorenzo Ciccione

**Affiliations:** 1 Cognitive Neuroimaging Unit, Commissariat à l’Energie Atomique (CEA), Institut National de la Santé et de la Recherche Médicale (INSERM), NeuroSpin Center, Université Paris-Saclay, 91191 Gif-sur-Yvette, France; 2 Collège de France, Université Paris-Sciences-Lettres (PSL), 11 Place Marcelin Berthelot, 75005 Paris, France

## Abstract

Concepts of exact number are often thought to originate from counting and the successor function, or from a refinement of the approximate number system (ANS). We argue here for a third origin: a shared language-of-thought (LoT) for geometry and arithmetic that involves primitives of repetition, concatenation, and recursive embedding. Applied to sets, those primitives engender concepts of exact integers through recursive applications of additions and multiplications. Links between geometry and arithmetic also explain the emergence of higher-level notions (squares, primes, etc.). Under our hypothesis, understanding a number means having one or several mental expressions for it, and their minimal description length (MDL) determines how easily they can be mentally manipulated. Several historical, developmental, linguistic, and brain imaging phenomena provide preliminary support for our proposal.

## The origins of number concepts

Since infancy, humans share with many animals an ANS in which neurons are tuned to approximate numerosities [1–4]. Infants also have precise representations of small numerosities (subitizing) and an early capacity to track the effects of adding or subtracting one element [5]. However, how do children move from these foundational abilities to an understanding of exact number – the knowledge that each integer *n* has a precise value that is distinct from other numbers? Prominent theories of the origins of number concepts attribute a major role either to counting and the successor function, or to a progressive refinement of the ANS, but both views have problems (discussed in Box 1). Furthermore, theories of numerical development should ultimately explain the extension of number concepts to very large numbers, zero, negative numbers, fractions, reals, squares, square roots, and imaginary numbers. It is difficult to see how a mere refinement of approximation or successor operations could explain the emergence of concepts such as 1000, 0, −2, ½, $\sqrt{2}$, or π.

In *The Number Sense* [6], one of us suggested that mathematics arose from ‘the specifically human competence for creating symbol systems … and connecting them to evolutionarily ancient nonverbal representations such as the quantity system’ [7]. Noah Goodman, Steven Piantadosi, Josh Tenenbaum, and many others have proposed that a LoT, an internal language capable of combining symbols into abstract mental programs, may underlie human conceptual learning [8–10]. This view has been applied to the development of some arithmetic [10–13] and geometric concepts [14–16] (Box 2). Building upon such prior work, we propose here that a shared LoT for geometry and arithmetic provides a plausible path towards higher-level mathematical concepts, possibly including complex mathematical constructions such as algebra and graphics [17–19].

The idea is simple. In the domain of geometry, we found that a language with recursive operations of shape concatenation, repetition, and nested composition generates the broad variety of signs and patterns which are attested throughout cultures since prehistory [16]. We show here that the same language, when applied to sets and their numerosities, may yield concepts of exact number and arithmetic. Concatenation yields addition, and nesting yields multiplication (e.g., two sets of 5). By naming some of these expressions (10 = 2 × 5) and adding them as new primitives, the syntax-of-sets engenders an infinite tree of exact arithmetic concepts [e.g., 65 = (6 × 10) + 5], while never losing their connection to concrete sets and their geometric layout. The proposed link between arithmetic and geometry fits with growing evidence that training with geometric patterns [20,21] or the abacus [22–24] can facilitate arithmetic development. In this opinion we first introduce the syntax-of-sets hypothesis, and then examine how it sheds light on past data and leads to novel predictions.

## A LoT for geometry, patterns, and sets

Since prehistory, cultures across the world have created geometric designs with lines, circles, zigzag, spirals, and their combinations. To explain this universal human propensity, which is lacking in other animals [14,25], we recently proposed that humans possess an inner LoT for geometry [16]. This recursive language, akin to the ‘turtle’ programming language Logo, allows them to combine lines and curves into composite shapes. For instance, the ‘square’ concept involves a mental program stating ‘repeat four times {trace a line; turn by 90°}’.

The LoT hypothesis stipulates that, when humans see a geometric shape or pattern, they attempt to infer the shortest program that draws it. The shorter this program, the easier it is to remember that shape. This is called the simplicity principle [26,27]: the simplest mental expressions, those with the smallest MDL, are easier to perceive and memorize. For geometry, this prediction was verified experimentally in several intruder detection and match-to-sample tasks [14,16]. The language itself is minimal: beyond tracing a line and turning, the only required control operations are repetition with variations, concatenation, and nesting (the latter, corresponding to a subprogram call, is necessary to represent, for instance, a square of circles or a line of squares). Remarkably, a similar language captures the psychological complexity of elementary musical or visual patterns made of two tones or two shapes [28,29]. For instance, the pattern xxOOxxOO has a short description (alternating pairs) and is simpler to memorize than, say, xOOOxxOx.

**Figure 1:**
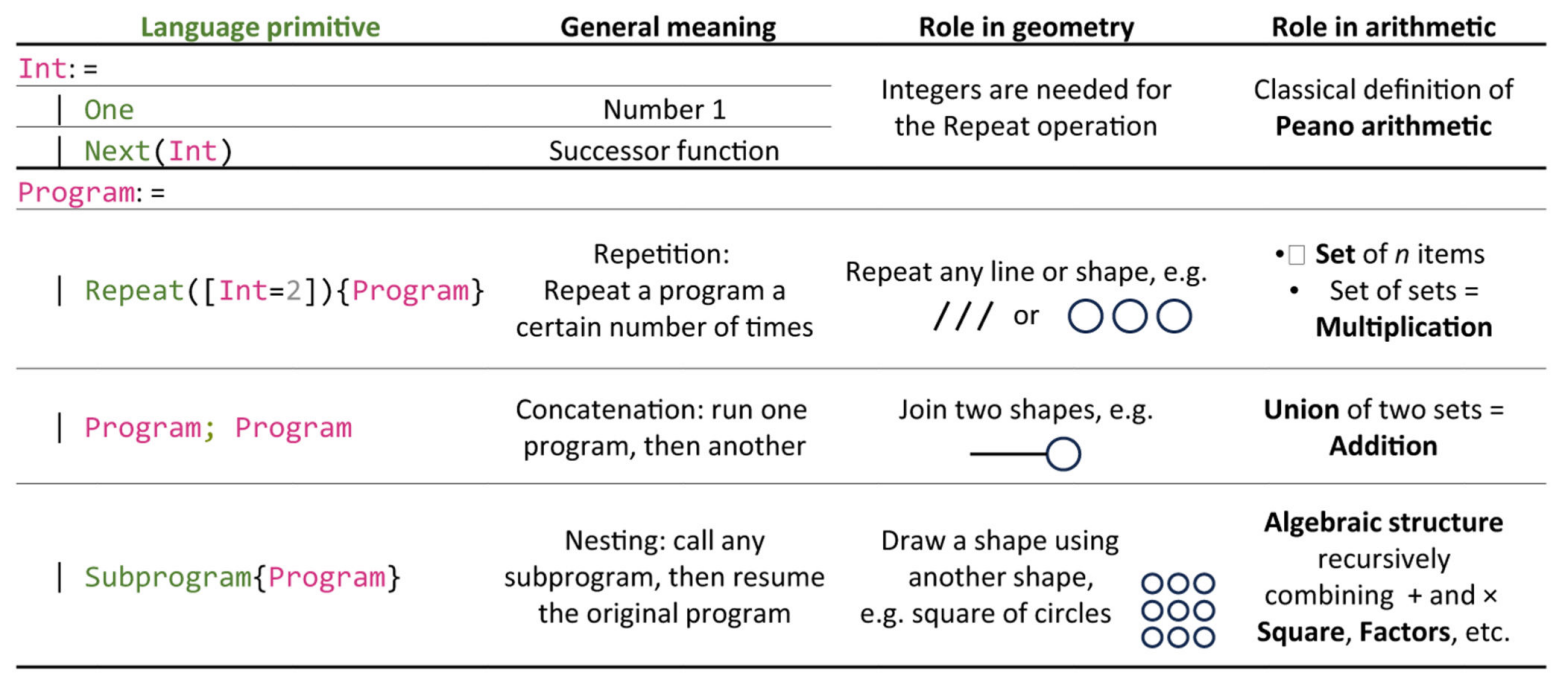
Proposed language for geometric shapes and its implications for arithmetic. The language postulates that geometric shapes are generated by only three recursive operations: repetition, concatenation, and nesting of simpler shapes. When applied to the primitive for drawing a single line (possibly with variations in curvature and acceleration), these operations can generate any regular geometric shape or pattern, and the simpler shapes (e.g., line, square, circle, square of circles, etc.) have the smallest minimum description length (MDL). When applied to a single identical item (e.g., a tally mark), the same operations can be interpreted as set formation (repetition of the mark *n* times), multiplication (repetition of a set), addition (union of disjoint sets), their recursive combinations (e.g., one item plus two sets of 3 = 7) and other regular geometrical arrangements (e.g., a square of *n* × *n* marks). We hypothesize that these operations lead to the algebra of integers, where the simplest numbers (e.g., even numbers, powers of 2) are those with the smallest MDL. Abbreviation: Int, integer.

We propose here that the same primitives may engender the basic concepts of mental arithmetic and explain their development and cultural emergence (Figure 1). From the start, drawing and numeration are linked. In prehistory, the earliest evidence for number concepts is tally marks: repeated dots, lines, or notches indicating the corresponding numerosity [30]. Such series of identical marks are among the simplest patterns in our proposed language-of-geometry because they only involve a repetition operation. However, the language also allows more complex combinations. First, using the concatenation operation, two rows of tally marks may be juxtaposed (as on the paleolithic Lebombo or Ishango bones). Such concatenation of *n* marks and *m* marks corresponds to the addition of their cardinals *n* + *m*. Second, the recursive character of the language allows one to form sets of sets (we use ‘recursion’ in the classic sense of the capacity to embed a mental representation within others of the same nature [31,32]). Thus, the language can express ‘*n* repetitions of *m* marks’, which corresponds to the multiplication *n* × *m* (as in the Lascaux prehistoric cave, where three groups of 2 dots appear behind a rhino). Third, these operations can be arbitrarily nested, thus forming a complete algebraic structure. Any number can be represented by nested + and × operations [e.g., 21 = (2 × 10) + 1].

In summary, repetition, concatenation, and recursion engender a language which renders intuitive not only the successor of a given number (+1) but also addition, multiplication, and their combinations. We call it a ‘syntax-of-sets’ because the expressions that it creates (e.g., II III) can be put into one-to-one correspondence with a set of objects of the corresponding numerosity, while abstracting away from their specific identity and focusing only on their internal structure (here a set of 2 plus a set of 3). A full account of set intuitions may require more primitives (e.g., intersection), but these seem to be the minimal ones.

The idea that recursive language-like structures play a key role in number development is not new (reviewed in Box 2). Our proposal is most similar to Guerrero and Park’s recent proposal [12] that addition and multiplication are the sole primitives that generate all integer concepts. Our specificity is that we come to this conclusion from our previous language-of-geometry proposal, thus offering a potential unification for the human sense of geometric and arithmetic patterns, and perhaps explaining why only those two operations arise early: in our Logo-like language for drawing shapes, there is no erase function that would be a potential equivalent of subtraction, even less a geometric equivalent of division.

Under our view, number concepts are inherently compositional and algebraic. Each number is internally represented by one or several expressions that connect it to other previously acquired concepts and can be ultimately traced to a very small number of primitives (1, +, and ×). How compactly they do so (their MDL) determines the ease of their development and comprehension.

## The construction of internal representations and its relation to external symbol systems

In our view, number concepts do not need to be constructed serially: having the concept 19 is not necessary before understanding 20. Instead, starting with the concept of 1, the repertoire of arithmetic concepts expands by recursive composition of previous concepts. The concept 2 emerges as the expression 1 + 1, then 3 emerges as 2 + 1, 4 as 2 + 2 or 2 × 2, etc.

Although this language can express an infinity of concepts, their exponential explosion is trimmed, in each culture, by focusing on a restricted set of expressions. For instance, in modern culture, the concept 10 (2 × 5) has a special status (‘base’) and becomes a new primitive for forming larger numbers. We assume that expressions must be automatized, compiled, and assigned a new symbol before they can become new primitives and enter higher-level compositions at reduced cost. Thus, different cultural choices may diverge in the MDL they assign to the same numbers, as was previously shown for geometric patterns [16]. In our culture, expressions in base 10 are easy, whereas base 60 was more natural for Babylonian mathematicians. Crucially, the deeper the pyramid of syntactic operations necessary to express the concept, the later it is predicted to appear over human phylogeny and ontogeny.

The proposed syntax-of-sets is an internal LoT and therefore differs from the linguistic numeration system [33,34]. Numerical concepts can be externalized using symbolic or linguistic representations, and these two types of systems (internal and external) support each other [35]. Nevertheless, in cultural evolution, our hypothesis is that the internal language came first and motivated the progressive invention of efficient external notation systems, either written or spoken. Undoubtedly, in present-day child development, the relationship is bidirectional. The evidence suggests that the availability of words, phrases, morphological markers, or symbols for a concept helps children to acquire the corresponding mental expressions [36–39]. For instance, in Chinese and other Asian languages, the verbal numeration system does not have teen and decade words, but expresses those concepts using a transparent base-10 syntax (e.g., 12 is ‘ten-two’ and 32 is ‘three-tens-two’). Children who speak Asian languages are less likely to err in tasks involving the understanding of place value [40,41] and the counting sequence [38,42]. When Chinese, Japanese, and Korean children are asked to construct numbers using blocks, they prefer using combinations of 10, whereas American children prefer using units [43]. These data reveal the important role played by natural language in scaffolding the mental representation of number concepts, but we propose that the format of this internal representation is expressions in the language of sets, not words.

## Historical and empirical evidence

A prerequisite of our theory is that recursive combinations of sets are conceptually available to all humans early on in development. We briefly point here to historical and developmental observations that support these hypotheses.

### Recursive combinations of addition and multiplication underlie numerical notations worldwide

An analysis of number notations worldwide [34] shows that, beyond mere counting, they all rely on recursive combinations of addition, multiplication, and occasionally subtraction, together with an emphasis on achieving maximal compactness (short MDL). In prehistory, cultures used successor-based notations using tally marks, adding a notch or a line for each counted item. However, beyond 3 or 4, when exact numerosity becomes difficult to perceive, humans broke their monotonicity by separating them into regular subgroups. For instance, Egyptian hieroglyphs denoted 5 as a row of 3 marks above another row of 2, and used similar groupings by 3 or by 4 for numbers up to 9, thus relying on set addition. To denote larger numbers, most notation systems relied on multiplication, typically by selecting one or a few standard set sizes (‘bases’) and counting them and their powers. Classical roman numerals, for instance, denoted five items by V, 10 items by X, 50 by L, 100 by C, 500 by D, and 1000 by M, thus representing for instance the number 137 as CXXXVII = 100 + 10 + 10 + 10 + 5 + 2 (a ‘cumulative-additive’ notation). Similarly, ancient Greek allocated 27 distinct letters to each unit 1–9, to each decade 10–90, and to each hundred 100–900, thus achieving a compact ‘ciphered-additive’ system where 137 = PΛZ. Combined addition and multiplication are even clearer in positional notations such as present-day Arabic numerals, where position indicates the multiplicand [e.g., 137 means (1 × 100) + (3 × 10) + 7], and in ‘multiplicative-additive’ systems, such as Chinese, where the multiplicand is explicitly denoted (137 reads 一百三十七 = ‘one-hundred three-tens seven’).

Recursion is also obvious in many notation systems. For instance, in ancient Greece, numbers larger than 999 were expressed by using a small comma-like mark known as a *hasta* to indicate multiplication by 1000 (B = 2; , B = 2000). As another example, the Babylonian system combined a base-10 cumulative-additive system for numbers 0–59, and a base-60 positional system beyond that, thus expressing 1434 as {[(2 × 10) + 3] × 60} + {(5 × 10) + 4}.

### Sets and their combinations are available early on in number development

A limited amount of developmental data supports our postulate that children quickly grasp how the numerosities of sets combine additively and multiplicatively. For instance, when children aged 4–6 years learned a new number word for three (‘gobi’), solely from exposure to phrases such as ‘one gobi house’ together with a corresponding set, they immediately generalized to ‘two gobi Xs’ according to a multiplicative meaning (2 × 3 = 6), thus revealing a capacity to represent nested sets [44]. In agreement with our hypothesis that the LoT for number is nonverbal, evidence suggests that even preverbal infants may understand sets of sets [45–48]. For instance, 14-month-olds successfully represent four objects in a box only when they are presented as two sets of 2 [45], thus using nested sets to decrease memory load and achieve greater numerical precision. Furthermore, they remember abstract set structure, not specific object identities [49]. At an even younger age, 5-month-olds discriminate four sets of 2 from two sets of 4, suggesting that they already attend to set structure, not merely overall numerosity [46]. Although the current data are compatible with chunking instead of recursion, these experiments offer excellent paradigms to evaluate whether, and at what age, children begin to understand sets and their combinations.

### Groupitizing points to the importance of set comprehension in arithmetic development

A recently discovered phenomenon, groupitizing, betrays how the syntax-of-sets facilitates the mental representation of numerosity. Groupitizing refers to the fact that, when asked to name the numerosity of a set of items, participants are faster and more accurate whenever the set can be easily decomposed into subsets [50–53]. For instance, the enumeration of nine is accelerated when presented as three groups of 3, whether grouped by spatial proximity or color, compared to random or additive arrangement (e.g., groups of 2, 3, and 4) [51]. Groupitizing appears at around first grade, and yields savings proportional to the child’s knowledge of elementary arithmetic [53]. In grades 3–8, groupitizing is a predictor of future mathematical achievement [52], suggesting that it may be a key step in arithmetic comprehension.

**Figure 2:**
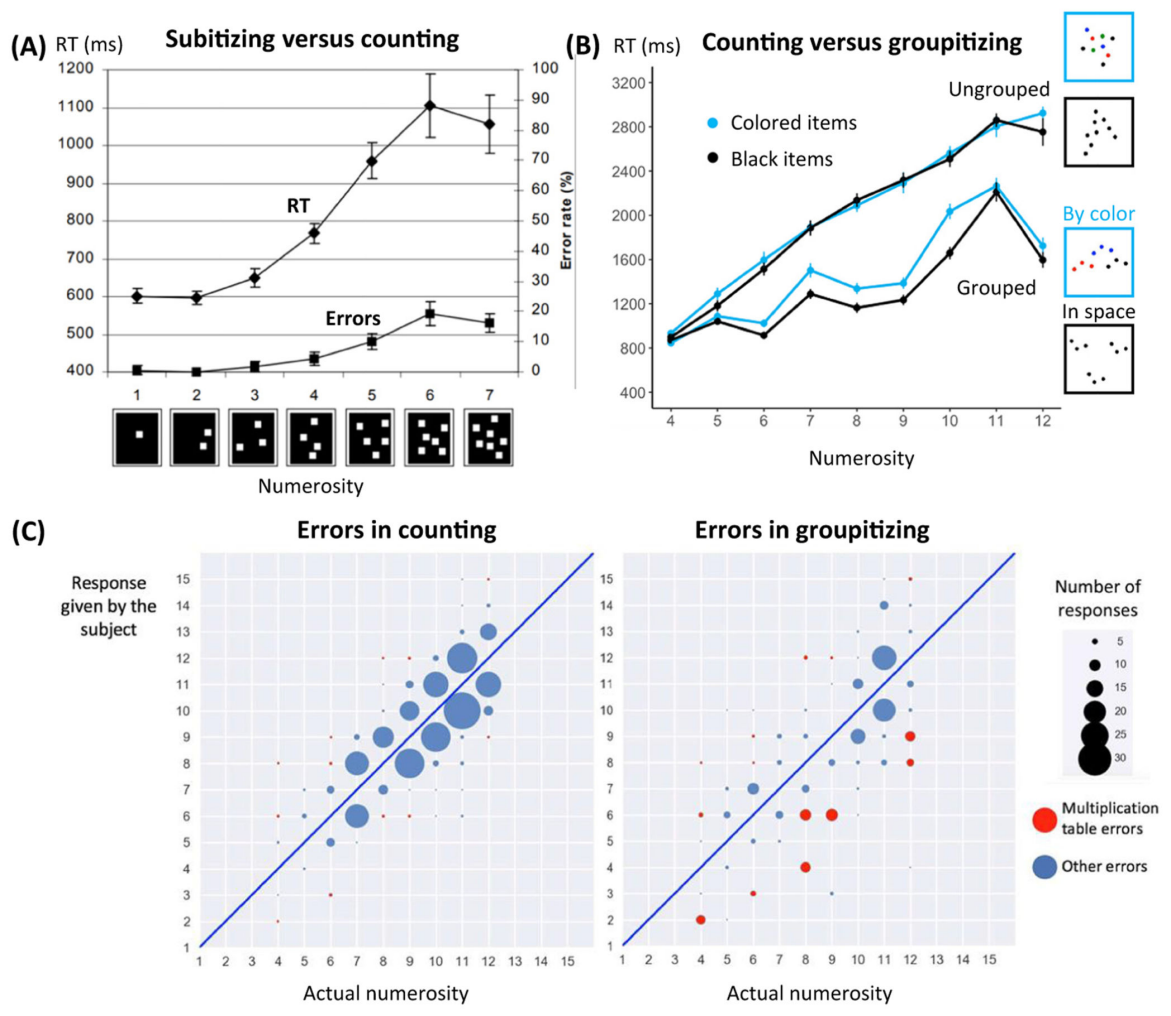
Groupitizing reflects the mental decomposition of numbers. Subitizing (A) is the fast evaluation of numerosities 1, 2, and 3. For larger sets, counting leads to a linear increase of naming time with numerosity (A and B). However, groupitizing is the empirical observation that substantial savings are observed if the set can be grouped into simple subsets (B). Note that the savings are larger for numbers that have a simpler decomposition into equal subsets, and the savings are therefore smaller for prime numbers 5, 7, and 11. Furthermore, enumeration errors (C) show a radically different pattern under enumeration and under groupitizing: counting errors typically occur around *n* ± 1, but groupitizing affords more exact numerosity naming, and rare errors reflect a slip in multiplication (e.g., responding 6 or even 4 to target 8). Panel (A) adapted, with permission, from [54]; panels (B,C) are redrawn from [51]. Abbreviation: RT, response time.

Groupitizing does not reduce to chunking because merely chunking a set into unequal groups (e.g., 9 = 2 + 3 + 4) yields barely any savings relative to an ungrouped situation [54] (Figure 2A). Larger savings arise from multiplication, namely sets of sets [51]. Consequently, as predicted by the MDL hypothesis, prime numbers, which cannot be divided into equal groups and have a more complex additive/multiplicative representation, show smaller savings than non-prime numbers (Figure 2B). Furthermore, during groupitizing, errors in naming numerosity are radically different than during estimation or counting: they no longer reflect numerical proximity, but syntactic resemblance. Thus, with three groups of 3, participants occasionally respond 6 (3 × 2), an error which almost never occurs with sets of 9 ungrouped items (Figure 2C) and betrays the syntax-of-sets.

## Empirical predictions and initial tests of the hypothesis

We point here to interesting consequences of our hypothesis, some of which have begun to receive empirical support.

### Skipping directly to large numbers

Our hypothesis predicts that multiplication and addition may allow humans to skip ahead to large numbers, for instance in correctly conceptualizing 100 as ‘10 sets of 10’ without necessarily mastering all previous numbers. Historically, some support may be found in the fact that some number notation systems relied on subtraction (e.g., Roman numerals 4 = IV = 5 − 1; 90 = XC = 100 − 10; also 8 = IIX and 80 = XXC [31]). In such systems, addition and multiplication were used to express large number concepts such as XX (20), and subtraction to return to smaller numbers such as XIX (19). Note that such cases also support the MDL hypothesis because subtraction shortens the notation (e.g., 90 = XC instead of LXXXX).

Psychologically, can one conceptualize larger numbers without mastering smaller ones? Although developmental evidence on this point remains to be collected, O’Shaughnessy *et al*. [55] tested Tsimane adults without formal education who were involved in selling products whose prices are often multiples of 5 pesos. Remarkably, although most could count up to 25 or more, they showed important difficulties in the addition of +1 (even for 1 + 1), whereas +5 was easier. In addition, small multiplications by 5 were easier for them than one-digit additions! This finding suggests that, through addition and multiplication, the Tsimane do grasp large numbers before smaller ones.

### Simplicity principle for number concepts

Our theory predicts that numbers with a shorter LoT expression should emerge earlier in development and be easier to conceptualize and manipulate in adulthood. Thus, within large numbers, those with simpler multiplicative decompositions should be easier to handle, and those with similar decompositions should be judged more similar. Limited evidence is currently available on this topic. Shepard [56] asked human participants to rate the conceptual similarity of numbers 0–9.

**Figure 3:**
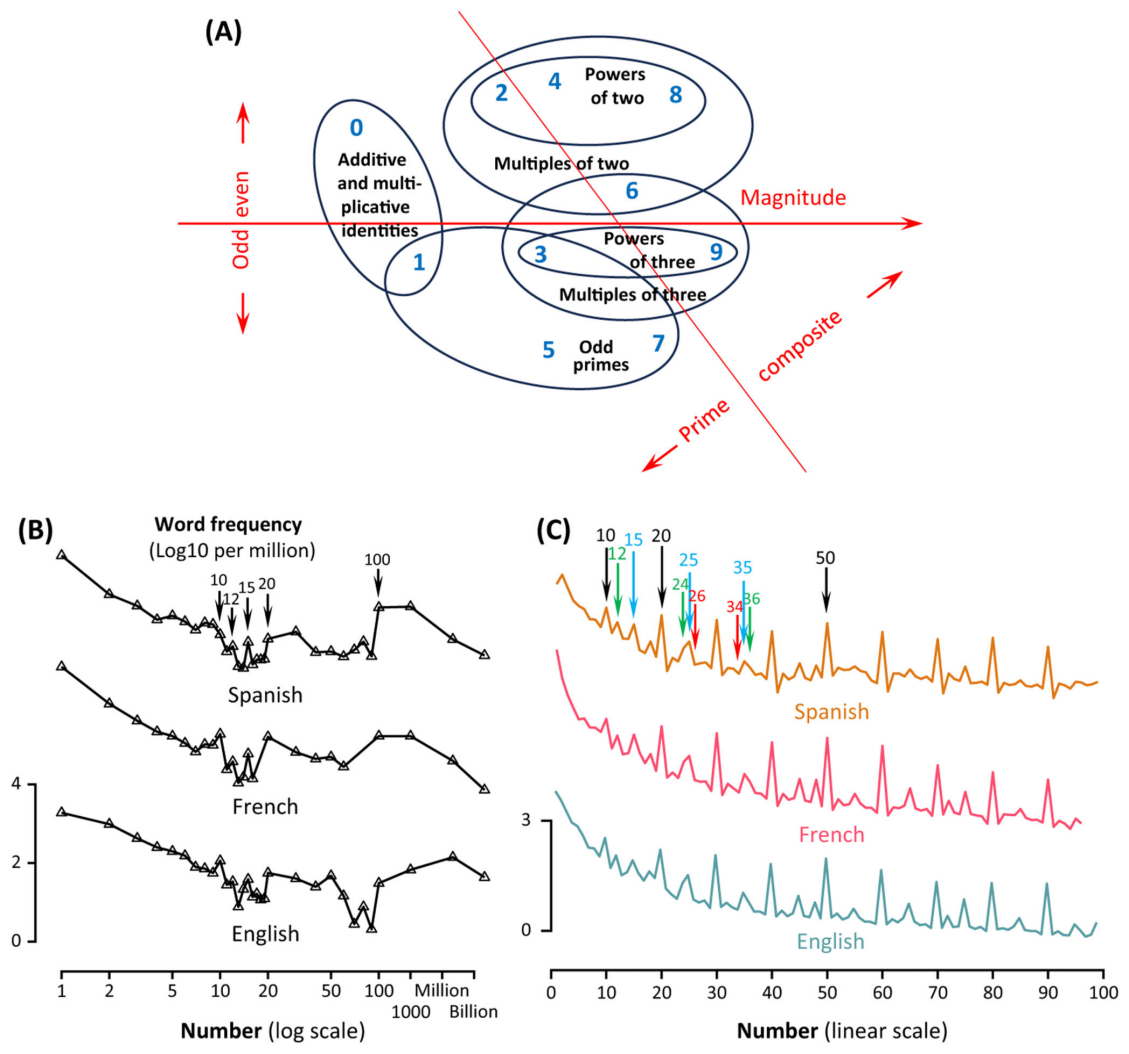
Evidence for a factorial representation of numbers in adults. (A) Data on the conceptual similarity of small integers. Adults rated the conceptual similarity of all pairs of Arabic digits (redrawn from [56]). The figure shows a 2D multidimensional scaling (MDS) projection of the 10 × 10 similarity matrix. Note that the main ordering is by magnitude (left to right), but also involves local groupings according to primality and to divisibility by 2 and by 3. (B,C) Data on the frequency of number words. In all languages tested, the frequency of number words decreases with their magnitude, an observation first made by Dehaene and Mehler in 1992 (panel B, replotted from data in [58]) and which can now be extended using the Google NGrams database (C). The figures show the Log10 frequency per million (curves for different languages are arbitrarily shifted vertically). There are replicable subpeaks for round numbers (decades, 12, 15) and any number with a large multiplicity of prime factors (e.g., 24 and 36 relative to 26 and 34). The most frequent numbers are those with the simplest algebraic expression, in other words the smallest minimum description length (MDL).

Multidimensional scaling revealed that, although magnitude was a primary determinant of similarity, other axes encoded more complex compositional properties, leading to separate groupings for powers of 2, powers of 3, and prime numbers (Figure 3). In a parity judgment task, similarly, even numbers and powers of 2 were responded to faster [57].

The frequency with which humans use number words offers another window into their mental representation. In all Western languages, the frequency of number *n* decreases as an inverse function of its magnitude, approximately as 1/*n*^2^, but with many exceptions, including additional frequency peaks at decades, powers of 10, and even of 12 and 15 [58]. The LoT hypothesis may explain this profile. Using modern databases such as Google NGrams, we obtained not only the frequency of individual numerals but also of combinations such as ‘twenty-four’. The resulting frequency profile is rich in regularities (Figure 3B). In English, French, Spanish, Italian, and German, numeral frequency exhibits reproducible subpeaks at multiples of 10, 5, 3, and 2 (Figure 3C). A multiple regression shows that Log(frequency) is not only proportional to Log(*n*) but is also positively related to the exponents of 2, 3, 5, and 10 in the factorization of *n*. Thus, frequency is higher for numbers with a simple decomposition into small factors – for instance, 24 (2^3^ × 3) is more frequent than 26 (2 × 13), and conversely 34 (2 × 17) is less frequent than 36 (2^2^ × 3^2^). These patterns fit with our assumption that the brevity of the derivation of a number, particularly using multiplication, determines its psychological simplicity and therefore its usage frequency. Indeed, the entire number-word frequency curve can be explained by a single unifying measure of MDL [59].

### Multiple expressions for the same numbers

Multiple mental expressions may exist for the same number (e.g., 6 = 5 + 1, 4 + 2, 2 × 3, and 3 × 2, etc.). We predict that the mastery of a fluid repertoire of different expressions for the same numbers, and an understanding of which expressions are equivalent, should be predictors of mathematical development in children. A larger repertoire should facilitate flexible problem solving (e.g., solving 9 + 7 as 10 + 6 by using 7 = 6 + 1 and 9 + 1 = 10). Under LoT, whether two expressions map onto the same number is not obvious. Because ‘*p* sets of *q* objects’ and ‘*q* sets of *p* objects’ are distinct expressions, our theory predicts, correctly, that commutativity should be unintuitive to children [60,61]. Pedagogically, we propose that an understanding of commutativity should be facilitated by linking arithmetic and geometry, for instance by showing that a 90° rotation makes the rectangles corresponding to *p* × *q* and *q* × *p* identical.

### No direct connection between LoT expressions and their magnitudes

Nothing in a LoT expression specifies the magnitude of a number: the expression 2 × 5, for instance, does not indicate whether it is larger, smaller, or equal to 3 × 4. We therefore predict a delayed development of the capacity to attribute the proper magnitude to large numerals. Indeed, even educated adults are miscalibrated and name a set of 50 dots as ‘thirty’ [62]. The algorithms that allow adults and children to translate between numerals and quantities [63–66], their development [67,68], their associative or structural nature [69], and the reason for their miscalibration should be studied empirically in greater depth.

### Distinct brain systems for approximate and exact number

The distinction between analog and LoT representations of number may also shed light on the cortical representation of number concepts. Our proposal predicts that a number such as 6 is represented by two distinct neural systems: (i) the analog number system, by a subset of neurons tuned to around 6; and (ii) the LoT, by a mental expression such as 5 + 1 or 2 × 3. However, if the task requires a magnitude (for instance for comparison or estimation), the latter expressions may be converted into a quantity. Thus, we predict that the overlap in cortical activity to symbolic and non-symbolic numbers should be task-dependent, with greater overlap in magnitude-dependent tasks, and greater separation for tasks that emphasize compositional manipulations, such as exact calculation or parity judgment. These considerations may explain seemingly contradictory brain imaging experiments that asked whether exact symbolic numbers and approximate non-symbolic numerosities converge onto the same neural code: some studies found overlap and cross-notation decoding, especially in tasks such as number comparison or estimation [70–73], whereas others found dissociations between symbolic and non-symbolic displays of the same numbers [2,74–82]. In addition, ANS in young children seems to be lateralized to the right intraparietal region [83,84], whereas education in arithmetic leads to a progressive shift towards the left inferior parietal region, which seems to host a more precise representation of exact number [73].

At present, little is known about the neural code underlying mental symbols and their compositionality. When adults encode natural integers, the left angular gyrus, left intraparietal sulcus, and left superior temporal sulcus seem to be particularly involved in compositional representations. Above all, the prefrontal cortex (PFC), particularly area 44d, seems to play a key role in forming the tree structures of geometrical, sequential, and numerical expressions [28,85,86]. A consistent developmental finding is greater PFC activation during numerical tasks in children [70,87,88]. LoT expressions may first be composed in PFC before becoming automatized and frozen into standalone primitives, possibly in parietal cortex and angular gyrus [89,90]. Interestingly, a recent developmental study [79] found that, between the ages of 5 and 8 years, the codes in parietal cortex for passively presented symbolic and non-symbolic numbers become increasingly dissimilar – exactly what would be predicted if children increasingly rely on representing numbers as LoT expressions and not merely as approximate magnitudes.

### A geometric path to higher-level arithmetic concepts

Most previous theories do not account for the human-specific expansion of arithmetic concepts beyond natural integers. If the human startup kit was limited to approximate numbers and the successor function, how would we develop concepts of square, prime, or negative numbers? However, brain imaging evidence suggests that higher-level mathematics, in educated adults and professional mathematicians, continues to encroach upon the same regions that initially encode geometric shapes and natural numbers [91–93]. The present view suggests that many high-level arithmetic concepts may emerge as compact mental expressions that link arithmetic and geometry – although they sometimes require extensions of the proposed language.

#### Squares, cubes, and other powers

The program for drawing a square is one of the simplest in our language-of-geometry [16]. Once the concept of number *n* is mastered, the program for drawing a square with *n* lines of *n* objects is easy (i.e., it has a small MDL). Historically, this is the origin of the concept of ‘square number’ (denoted *n*^2^). Cubes may be similarly defined, and exponentiation arises by iteration of the same idea (*n*^p^ = multiplying the number *n* by itself *p* times).

#### Even and odd numbers

In the proposed language, rectangles of dots also have short expressions. Furthermore, their nested expression (*p* sets of *q* items) evokes a product (*n* = *p* × *q*). Thus, the language draws attention to products of small integers and, because duplication is the default repetition, multiplication by 2 (doubling) is most natural. The corresponding numbers are given a name: even numbers (*n* = 2q). Those that cannot be put in this form are called odd, as indeed their description is longer (*n* = 2q + 1).

#### Factorization and prime numbers

Thinking about rectangles leads to a natural question: can any number be framed as a rectangular box? This is called factorizing. Some numbers, such as 5, 7, and 11, cannot (if one excludes a trivial box with a side of 1): these are called prime numbers. Thus, the theory can explain the emergence of these concepts, and they remain highly productive sources of mathematical developments to this day.

#### Inverse operations

Concepts of negative numbers, square roots, fractions, or complex numbers are not included in the present language, but may become accessible with a simple extension: the inclusion of expressions such as ‘the number *n* such that…’ (as available for instance in logic programming languages such as Prolog). For instance, ‘the number *n* such that *n* + *p* = *q’* is a compact expression for *n* = *q* − *p* (subtraction). Adding such a capacity to ‘solve for *n’* allows many new concepts to be defined, such as square root (the number *n* such that *n* × *n* = *p*), negative number (*n* such that *n* + *p* = 0), fraction (*n* such that *n* × *p* = q), and even imaginary and complex numbers (*n* such that *n* × *n* = −1). Do such numbers ‘exist’? In our framework, this question is immaterial. Because they are well-formed expressions in the LoT, they are ‘thinkable’, and, ultimately, if they play productive, non-contradictory roles, they are included in the mathematical edifice [94].

#### Negation and infinity

Another useful addition to our language is negation, an elementary operator that is present in all languages [95] and is available to infants [96–98]. Negation is necessary to define odd numbers (‘not even’), prime numbers (‘not factorizable’), and many other concepts such as ‘infinite’. The latter arises naturally to preschoolers when they ask themselves whether there is a number larger than all others [99,100].

#### Number lines, measurement, and the links between arithmetic and geometry

In our theory, a single language captures continuous figures (e.g., a square) and discrete figures (e.g., a square of dots). Indeed, numbers are figures – or, in other words, numbers and geometric figures – arise from similar expressions in the LoT. It comes as no surprise, therefore, that number–space links are omnipresent in mathematics. For instance, a segment of fixed length can be repeated *n* times to form a longer line – thus leading to the concept of measurement in which lines are graduated by integers, their multiples, and their fractions. However, some lines are impossible to measure together, and are thus ‘incommensurable’. The Greeks discovered that the diagonal of the unit square, of length $\sqrt{2}$ , cannot be expressed as a fraction of its side. This finding led to one of the most productive struggles in mathematics and, ultimately, to the invention of real numbers. We propose that all fields of mathematics arise from such a search for coherence and completeness in the internal LoT.

### Concluding remarks

Our proposal is complementary to analog and successor theories of number development. Undoubtedly, analog representations of magnitudes contribute to early number sense, and so do counting and the successor concept. Indeed, +1 is one of the simplest expressions in our language. However, we and others propose that exact numbers arise from a richer compositional system – a LoT that is capable of generating recursive expressions with + and ×, and directly parallels similar expressions with sets of marks and geometric shapes. Our proposal can explain the tight links between geometry and arithmetic in the history of mathematics, and we suggest that equally tight links have arisen during the development of mathematics. Our proposal is falsifiable: for example, evidence could be found showing that groupitizing is a solely perceptual or attentional phenomenon that is devoid of any conceptual component, or that geometry and arithmetic are sustained by dissociable systems. So far, to the best of our knowledge, the opposite is found, both in development and in adulthood.

Many questions arise (see Outstanding questions). Most crucially, if a mathematical LoT is available early on, why are mathematical concepts so slow to develop? Why does groupitizing arise during primary education, and concepts of parity, square, and prime number typically only emerge in secondary school? Although the question is open, several tentative answers can be proposed. First, compared to the basic concepts of line or curve, the program for a square is already a significant geometric construction, and there is still a significant jump from there to the generic concept of ‘square number’. A child may recognize that a particular array forms a square without understanding that some numbers can be put in this form whereas others cannot. Under our view, she must first acquire many expressions for specific numbers (e.g., 4 = 2 × 2, 6 = 3 × 2), and it will take time for this repertoire to become large enough to support groupitizing, and subsequently the higher-level expressions that characterize square or prime numbers. Furthermore, children receive far less stimulation in geometry and sets than in natural language. Indeed, the present ideas have broad pedagogical consequences. They predict that presenting children with concrete geometric and set manipulations, such as asking them to arrange items into *n* rows of *n* (a square), should facilitate the emergence of arithmetic concepts.

## Box 1 Two major theories for the origins of arithmetic, and their problems

Many theories have been proposed for the origins of symbolic arithmetic in humans. We briefly mention two major classes. A prominent view proposes that human arithmetic is founded upon an approximate number system (ANS). Although this system is present in many other animal species such as monkeys or crows [3,101], it is coarse and continuous, and therefore cannot reliably discriminate between exact numbers such as 17 and 18. In humans, however, this system is refined under the influence of learning written and spoken numerals [6,101].

The ANS undoubtedly plays an important role in numerical cognition throughout life. Two behavioral signatures of the ANS – the distance effect, whereby distant numbers are easier to discriminate than close numbers, and Weber’s law, by which this effect scales up for larger numbers – are ubiquitous whenever humans compare or approximate numbers, in infancy [102], preschool [103], and adulthood [6,104,105]. Adults use the ANS to estimate prices [106] or reject a calculation as grossly false [104,107]. However, there is growing evidence against the ANS hypothesis as a sufficient foundation for human arithmetic [12,82,108]. Although the precision of the ANS improves with age and education [109], it seems to be education in formal arithmetic that causes this refinement rather than the converse [110]. Indeed, a large-scale study of approximation training did not lead to any long-term improvements in symbolic arithmetic [111], and other findings to the contrary [112] do not seem to be replicable [113,114]. Even in educated adults, number-to-quantity mappings are imprecise, uncalibrated, and off by a wide margin [62], and exact and approximate arithmetic are neurologically dissociable [104,115,116]. Thus, the ANS underlies human intuitions of approximate quantities, not the emergence of exact arithmetic [6].

An alternative proposal is that arithmetic is founded upon an innate understanding of number 1 and the successor function [117–119]. All natural numbers would derive from number 1 through a single primitive successor operator S, such that 2 = S(1), 3 = S[S(1)], and so on. In some theories, an internal successor function generates distinct mental symbols for each consecutive discrete numerosity [120]. In others, children first learn the succession of external count words and slowly map the first three of them onto pre-existing object-file representations for 1, 2, and 3 [121,122], then suddenly induce that all number words map onto distinct discrete number concepts [13,118,123,124]. Mastering the successor function as a productive counting rule seems to help in understanding that numbers can be infinite [125]. Several arguments and experimental results argue against the successor view, however [12,102,108,126]. Within their counting range, many children cannot tell the ordinal position of a number, the successor of a given number, or which of two numbers is larger [127]. Conversely, children may map number words onto approximate magnitudes and sometimes exact numerosities up to 5 before mastering counting [108]. Furthermore, they may have conceptual knowledge of numbers beyond their counting list, including very large numbers such as ‘hundred’, ‘thousand’, or ‘million’ [67]. Speakers of Tsimane may understand the addition of number 5 and its multiples, but not of lower numbers [55]. Finally, some numbers seem to be psychologically simpler and more frequent than their predecessors (e.g., 20 vs. 19), in direct contradiction to the idea that each number builds solely upon its predecessor. Thus, verbal counting knowledge is neither necessary nor sufficient for arithmetic understanding. Indeed, it is hard to see how the successor function alone could account for large numbers such as 1000 or other high-level arithmetic concepts (multiplication, squares, primes, etc.).

## Box 2 Previous proposals relating language, language-of-thought, and the acquisition of number concepts.

Much previous work has emphasized the key role of linguistic and recursive structures in the acquisition of number concepts. Hurford [33] first developed a recursive formal grammar for numerals in various languages, based on three recursive rules (for English):

R1. A number can be a terminal (a single tally mark ‘/’) or a phrase, followed by an optional number.
R2. A phrase is a number followed by a multiplier.
R3. A multiplier is either ‘ten’ (realized as ‘-teen’ or ‘-ty’) or a number followed by a multiplier.

Those syntactic rules have semantic interpretations: they incorporate the successor function (R1), concatenation as addition (R1), phrase structure as multiplication (R2), and even powers of 10 as ‘multiplication *n* times’ (R3). They allow numerals to be described as tree structures with recursive calls to addition and multiplication (see Figure I for an example).

Although tightly related to the present proposal, Hurford’s theory differs in its focus on number words and their variability across languages, rather than on a theory of internal concepts. Spelke [108], following Chomsky, proposed that children acquire natural number concepts through ‘mastery of the generative rules of language’. Exposed to phrases such as ‘a dog and a cat’ or ‘two animals’, children would map them onto their object-tracking system, thus allowing them to understand the meaning of the first three number words. More complex phrases such as ‘three cows and two more’ or ‘two groups of three puppets’ allow them to induce larger exact numbers. Although close to ours, this proposal differs in supposing that integers arise from natural language, not from an internal LoT that is shared with geometry. Two problems are that (i) the proposal presupposes that the child is immersed in a language for numerals, without explaining how this language arose in the first place; and (ii) brain imaging shows that number and geometry activate overlapping brain areas which are distinct from language areas [28,85,86,91–93,128], even early on in development [83].

Within the LoT perspective propounded by Tenenbaum, Goodman, and colleagues [8,124], Piantadosi proposed an explicit formal language based on lambda calculus for the mental construction of exact numbers [13,124], thus fleshing out Carey’s previous bootstrapping proposal [119,123]. Piantadosi’s model nicely captures how children slowly learn that each numerosity maps onto a specific count word [118,122,123]. By further assuming that languages optimize the tradeoff between complexity and informativeness, one can infer which primitives are most likely given the variety of numeral systems worldwide. Denić and Szymanik [11] suggested that addition, subtraction, multiplication, division, and the numbers 1, 2, 5, and 10 suffice – but given that 5 and 10 are unlikely to be primitives available to infants, the proposal remains incomplete as a full theory of number development.

In animal cognition, Gallistel [129] proposed that the representations of approximate numbers and other continuous quantities must enter into an algebraic structure that combines addition and multiplication. The proposal was extended to exact number by Leslie *et al*. [130], although only in a minimal model with the primitives 1 and successor.

The two research groups whose work is most closely related to the present one are Halberda and Feigenson [48], who see the recursive concept of ‘set’ as the core of natural numbers and provide experimental evidence on infant set perception [45,47,131], and Guerrero and Park [12], who argue that number acquisition involves understanding the ‘generative rules that govern the system of natural numbers’, including successor, addition, and multiplication, and study empirically the development of the embedded structure of numbers [36]. These proposals, however, do not emphasize the important role of MDL, nor the links between geometry, space, and arithmetic. For example, spatial working memory and finger manipulation tasks have been shown to interfere with arithmetic performance even in adults [132,133], and spatial and arithmetic skills are tightly linked in development [134].

## Outstanding questions

What is the relation between the language of sets and natural language? These languages must be linked because arithmetic concepts can be expressed in natural language. During development, does natural language serve as a compositional ‘glue’ that brings together earlier core knowledge systems? Or is it only a pragmatic device, a temporary scaffold or catalyst that draws attention to relevant conceptual combinations, thus reducing the combinatorial explosion inherent to recursive systems?

Can the LoT approach be extended to other mathematical constructions beyond number and geometry, such as algebra or graphics?

How are compositional languages implemented in brain circuitry? Can artificial intelligence model how networks of neurons implement recursive symbolic structures?

What are the roles of the two hemispheres in the construction of mathematics? Does the lateralization of natural language influence the lateralization of mathematics, as suggested by their colateralization?

Why is the acquisition of mathematics so slow and difficult compared to language acquisition?

Can the acquisition of mathematics be facilitated by focusing the attention of children on the compositional origins of mathematical concepts?

Are there reliable individual differences in the richness of the repertoire of numerical expressions, and do they depend on mathematics education and achievement?

How do the primitives of the proposed LoT become instantiated in human language and behavior? Is externalization of such primitives (through symbolic or linguistic representations) necessary for arithmetic development?

## Highlights

A single language-of-thought (LoT) can serve as a foundation for the concepts of geometric figures, sets, and exact numbers.

Concatenation and recursion (sets of sets) provide intuitive foundations for how integers combine with + and × operations.

Understanding an exact number entails having one or several mental expressions that connect it to other numbers (e.g., 6 = three sets of 2).

Minimum description length (MDL) – the length of the shortest expression for a number – determines the ease of its acquisition and manipulation.

The frequency of numbers in natural language reflects their mental composition.

Links between geometry and arithmetic explain the emergence of number concepts such as ‘square number’.

## References

[1] Gennari G (2023). Spontaneous supra-modal encoding of number in the infant brain. Curr Biol.

[2] Kutter EF (2018). Single neurons in the human brain encode numbers. Neuron.

[3] Nieder A (2021). Neuroethology of number sense across the animal kingdom. J Exp Biol.

[4] Wagener L (2018). Neurons in the endbrain of numerically naive crows spontaneously encode visual numerosity. Curr Biol.

[5] Wynn K (1992). Addition and subtraction by human infants. Nature.

[6] Dehaene S (2011). The Number Sense.

[7] Dehaene S (2001). Author’s response: is number sense a patchwork?. Mind Lang.

[8] Goodman ND (2014). Concepts in a Probabilistic Language of Thought.

[9] Tenenbaum JB (2011). How to grow a mind: statistics, structure, and abstraction. Science.

[10] Yang Y, Piantadosi ST (2022). One model for the learning of language. Proc Natl Acad Sci U S A.

[11] Denić M, Szymanik J (2022). Reverse-engineering the language of thought: a new approach.

[12] Guerrero D, Park J (2023). Arithmetic thinking as the basis of children’s generative number concepts. Dev Rev.

[13] Piantadosi ST (2023). The algorithmic origins of counting. Child Dev.

[14] Dehaene S (2022). Symbols and mental programs: a hypothesis about human singularity. Trends Cogn Sci.

[15] Sablé-Meyer M (2021). Sensitivity to geometric shape regularity in humans and baboons: a putative signature of human singularity. Proc Natl Acad Sci U S A.

[16] Sablé-Meyer M (2022). A language of thought for the mental representation of geometric shapes. Cogn Psychol.

[17] Ciccione L, Dehaene S (2021). Can humans perform mental regression on a graph? Accuracy and bias in the perception of scatterplots. Cogn Psychol.

[18] Ciccione L (2023). Trend judgment as a perceptual building block of graphicacy and mathematics, across age, education, and culture. Sci Rep.

[19] Ciccione L (2022). Analyzing the misperception of exponential growth in graphs. Cognition.

[20] Zippert EL (2019). Not just IQ: patterning predicts preschoolers’ math knowledge beyond fluid reasoning. J Cogn Dev.

[21] Zippert EL (2020). Finding patterns in objects and numbers: repeating patterning in pre-K predicts kindergarten mathematics knowledge. J Exp Child Psychol.

[22] Hyde DC (2021). Testing the role of symbols in preschool numeracy: an experimental computer-based intervention study. PLoS One.

[23] Stigler JW (1984). Mental abacus: the effect of abacus training on Chinese children’s mental calculation. Cogn Psychol.

[24] Barner D (2016). Learning mathematics in a visuospatial format: a randomized, controlled trial of mental abacus instruction. Child Dev.

[25] Saito A (2014). The origin of representational drawing: a comparison of human children and chimpanzees. Child Dev.

[26] Chater N, Vitányi P (2003). Simplicity: a unifying principle in cognitive science?. Trends Cogn Sci.

[27] Feldman J (2003). The simplicity principle in human concept learning. Curr Dir Psychol Sci.

[28] Al Roumi F (2023). Brain-imaging evidence for compression of binary sound sequences in human memory. eLife.

[29] Planton S (2021). A theory of memory for binary sequences: evidence for a mental compression algorithm in humans. PLoS Comput Biol.

[30] d’Errico F (2018). From number sense to number symbols. An archaeological perspective. Philos Trans R Soc B Biol Sci.

[31] Johnson-Laird PN (2022). Recursion in programs, thought, and language. Psychon Bull Rev.

[32] Liao DA (2022). Recursive sequence generation in crows. Sci Adv.

[33] Hurford JR (1975). The Linguistic Theory of Numerals.

[34] Chrisomalis S (2010). Numerical Notation: A Comparative History.

[35] Zhang J, Norman DA (1995). A representational analysis of numeration systems. Cognition.

[36] Guerrero D (2020). Is thirty-two three tens and two ones? The embedded structure of cardinal numbers. Cognition.

[37] Miller K (1995). Preschool origins of cross-national differences in mathematical competence: the role of number-naming systems. Psychol Sci.

[38] Schneider RM (2020). Do children use language structure to discover the recursive rules of counting?. Cogn Psychol.

[39] Le Corre M (2016). Numerical morphology supports early number word learning: evidence from a comparison of young Mandarin and English learners. Cogn Psychol.

[40] Miura IT, Okamoto Y (1989). Comparisons of U.S. and Japanese first graders’ cognitive representation of number and understanding of place value. J Educ Psychol.

[41] Miura IT (1993). First graders’ cognitive representation of number and understanding of place value: cross-national comparisons – France, Japan, Korea, Sweden, and the United States. J Educ Psychol.

[42] Miller KF, Stigler JW (1987). Counting in Chinese: cultural variation in a basic cognitive skill. Cogn Dev.

[43] Miura IT (1988). Effects of language characteristics on children’s cognitive representation of number: cross-national comparisons. Child Dev.

[44] Cheung P, Dale M (2016). A cross-linguistic investigation on the acquisition of complex numerals.

[45] Feigenson L, Halberda J (2004). Infants chunk object arrays into sets of individuals. Cognition.

[46] Wynn K (2002). Enumeration of collective entities by 5-month-old infants. Cognition.

[47] Zosh JM (2011). Memory for multiple visual ensembles in infancy. J Exp Psychol Gen.

[48] Halberda J, Feigenson L (2008). Set representations required for the acquisition of the ‘natural number’ concept. Behav Brain Sci.

[49] Kibbe MM, Feigenson L (2016). Infants use temporal regularities to chunk objects in memory. Cognition.

[50] Anobile G (2020). ‘Groupitizing’: a strategy for numerosity estimation. Sci Rep.

[51] Ciccione L, Dehaene S (2020). Grouping mechanisms in numerosity perception. Open Mind.

[52] Guillaume M (2023). Groupitizing reflects conceptual developments in math cognition and inequities in math achievement from childhood through adolescence. Child Dev.

[53] Starkey GS, McCandliss BD (2014). The emergence of ‘groupitizing’ in children’s numerical cognition. J Exp Child Psychol.

[54] Piazza M (2003). Single-trial classification of parallel pre-attentive and serial attentive processes using functional magnetic resonance imaging. Proc R Soc Lond B.

[55] O’Shaughnessy DM (2023). Diverse mathematical knowledge among indigenous Amazonians. Proc Natl Acad Sci U S A.

[56] Shepard RN (1975). The internal representation of numbers. Cogn Psychol.

[57] Dehaene S (1993). The mental representation of parity and numerical magnitude. J Exp Psychol Gen.

[58] Dehaene S, Mehler J (1992). Cross-linguistic regularities in the frequency of number words. Cognition.

[59] Pajot M (2025). The compositional nature of number concepts: insights from number frequencies. PsyArXiv.

[60] Amalric M (2023). Do school-age children learn that 2 × 3 = 3 × 2 relying on previous intuitions?.

[61] Baroody AJ, Baroody AJ, Dowker A (2003). The Development of Arithmetic Concepts and Skills: Constructing Adaptive Expertise.

[62] Izard V, Dehaene S (2008). Calibrating the mental number line. Cognition.

[63] Dotan D (2021). Serial and syntactic processing in the visual analysis of multi-digit numbers. Cortex.

[64] Dotan D, Dehaene S (2013). How do we convert a number into a finger trajectory?. Cognition.

[65] Dotan D, Dehaene S (2016). On the origins of logarithmic number-to-position mapping. Psychol Rev.

[66] Odic D (2015). Children’s mappings between number words and the approximate number system. Cognition.

[67] Cheung P, Ansari D (2023). A million is more than a thousand: children’s acquisition of very large number words. Dev Sci.

[68] Siegler RS, Opfer JE (2003). The development of numerical estimation: evidence for multiple representations of numerical quantity. Psychol Sci.

[69] Sullivan J, Barner D (2014). Inference and association in children’s early numerical estimation. Child Dev.

[70] Cantlon JF (2009). The neural development of an abstract concept of number. J Cogn Neurosci.

[71] Eger E (2009). Deciphering cortical number coding from human brain activity patterns. Curr Biol.

[72] Holloway ID, Ansari D (2009). Developmental specialization in the right intraparietal sulcus for the abstract representation of numerical magnitude. J Cogn Neurosci.

[73] Piazza M (2007). A magnitude code common to numerosities and number symbols in human intraparietal cortex. Neuron.

[74] Bankson BB (2019). Whole-brain MEG decoding of symbolic and non-symbolic number stimuli reveals primarily format-dependent representations. BioRxiv.

[75] Bulthé J (2014). Format-dependent representations of symbolic and non-symbolic numbers in the human cortex as revealed by multi-voxel pattern analyses. NeuroImage.

[76] Castelli F (2006). Discrete and analogue quantity processing in the parietal lobe: a functional MRI study. Proc Natl Acad Sci U S A.

[77] Kadosh Cohen (2007). Notation-dependent and -independent representations of numbers in the parietal lobes. Neuron.

[78] Lyons IM (2015). Qualitatively different coding of symbolic and nonsymbolic numbers in the human brain. Hum Brain Mapp.

[79] Nakai T (2023). Cortical representations of numbers and nonsymbolic quantities expand and segregate in children from 5 to 8 years of age. PLoS Biol.

[80] Sokolowski HM (2021). Symbols are special: an fMRI adaptation study of symbolic, nonsymbolic, and non-numerical magnitude processing in the human brain. Cereb Cortex Commun.

[81] Sokolowski HM (2017). Common and distinct brain regions in both parietal and frontal cortex support symbolic and nonsymbolic number processing in humans: a functional neuroimaging meta-analysis. NeuroImage.

[82] Wilkey ED, Ansari D (2020). Challenging the neurobiological link between number sense and symbolic numerical abilities. Ann N Y Acad Sci.

[83] Izard V (2008). Distinct cerebral pathways for object identity and number in human infants. PLoS Biol.

[84] Ansari D (2008). Effects of development and enculturation on number representation in the brain. Nat Rev.

[85] Sablé-Meyer M (2025). A geometric shape regularity effect in the human brain. BioRxiv.

[86] Wang L (2019). Representation of spatial sequences using nested rules in human prefrontal cortex. NeuroImage.

[87] Ansari D (2005). Neural correlates of symbolic number processing in children and adults. Neuroreport.

[88] Kucian K (2008). Development of neural networks for exact and approximate calculation: a fMRI study. Dev Neuropsychol.

[89] Hung Y-H (2015). Neural correlates of merging number words. Neuroimage.

[90] Vogel SE (2015). Developmental specialization of the left parietal cortex for the semantic representation of Arabic numerals: an fMR-adaptation study. Dev Cogn Neurosci.

[91] Amalric M, Dehaene S (2016). Origins of the brain networks for advanced mathematics in expert mathematicians. Proc Natl Acad Sci U S A.

[92] Amalric M, Dehaene S (2019). A distinct cortical network for mathematical knowledge in the human brain. NeuroImage.

[93] Amalric M, Dehaene S (2017). Cortical circuits for mathematical knowledge: evidence for a major subdivision within the brain’s semantic networks. Philos Trans R Soc Lond Ser B Biol Sci.

[94] Gowers T (2002). Mathematics: A Very Short Introduction.

[95] Tettamanti M (2008). Negation in the brain: modulating action representations. Neuroimage.

[96] Cesana-Arlotti N (2018). Precursors of logical reasoning in preverbal human infants. Science.

[97] de Carvalho A (2021). ‘Look! It is not a bamoule!’: 18- and 24-month-olds can use negative sentences to constrain their interpretation of novel word meanings. Dev Sci.

[98] Ekramnia M (2021). Disjunctive inference in preverbal infants. iScience.

[99] Falk R (2010). The infinite challenge: levels of conceiving the endlessness of numbers. Cogn Instr.

[100] Monaghan J (2001). Young peoples’ ideas of infinity. Educ Stud Math.

[101] Piazza M (2010). Neurocognitive start-up tools for symbolic number representations. Trends Cogn Sci.

[102] Spelke ES (2022). What Babies Know: Core Knowledge and Composition.

[103] Gilmore CK (2007). Symbolic arithmetic knowledge without instruction. Nature.

[104] Dehaene S (1999). Sources of mathematical thinking: behavioral and brain-imaging evidence. Science.

[105] Moyer R, Landauer T (1967). Time required for judgements of numerical inequality. Nature.

[106] Dehaene S, Marques JF (2002). Cognitive Euroscience: scalar variability in price estimation and the cognitive consequences of switching to the Euro. Q J Exp Psychol.

[107] Ashcraft MH, Battaglia J (1978). Cognitive arithmetics: evidence for retrieval and decision processes in mental addition. J Exp Psychol Hum Learn Mem.

[108] Spelke ES (2017). Core knowledge, language, and number. Lang Learn Dev.

[109] Piazza M (2010). Developmental trajectory of number acuity reveals a severe impairment in developmental dyscalculia. Cognition.

[110] Piazza M (2013). Education enhances the acuity of the non-verbal approximate number system. Psychol Sci.

[111] Dillon MR (2017). Cognitive science in the field: a pre-school intervention durably enhances intuitive but not formal mathematics. Science.

[112] Park J, Brannon EM (2013). Training the approximate number system improves math proficiency. Psychol Sci.

[113] Szkudlarek E (2021). Failure to replicate the benefit of approximate arithmetic training for symbolic arithmetic fluency in adults. Cognition.

[114] Szűcs D, Myers T (2017). A critical analysis of design, facts, bias and inference in the approximate number system training literature: a systematic review. Trends Neurosci Educ.

[115] Lemer C (2003). Approximate quantities and exact number words: dissociable systems. Neuropsychologia.

[116] Dehaene S, Cohen L (1991). Two mental calculation systems: a case study of severe acalculia with preserved approximation. Neuropsychologia.

[117] Izard V (2008). Exact equality and successor function: two key concepts on the path towards understanding exact numbers. Philos Psychol.

[118] Carey S, Barner D (2019). Ontogenetic origins of human integer representations. Trends Cogn Sci.

[119] Carey S (1998). Knowledge of number: its evolution and ontogeny. Science.

[120] Gallistel CR, Gelman R, Kessen W (1991). Memories, Thoughts, and Emotions: Essays in Honor of George Mandler.

[121] Sarnecka BW, Lee MD (2009). Levels of number knowledge during early childhood. J Exp Child Psychol.

[122] Wynn K (1992). Children’s acquisition of the number words and the counting system. Cogn Psychol.

[123] Carey S (2009). The Origins of Concepts.

[124] Piantadosi ST (2012). Bootstrapping in a language of thought: a formal model of numerical concept learning. Cognition.

[125] Chu J (2020). Counting to infinity: does learning the syntax of the count list predict knowledge that numbers are infinite?. Cogn Sci.

[126] Cheung P (2017). To infinity and beyond: children generalize the successor function to all possible numbers years after learning to count. Cogn Psychol.

[127] Davidson K (2012). Does learning to count involve a semantic induction?. Cognition.

[128] Fedorenko E, Varley R (2016). Language and thought are not the same thing: evidence from neuroimaging and neurological patients. Ann N Y Acad Sci.

[129] Gallistel CR (1990). The Organization of Learning.

[130] Leslie AM (2008). The generative basis of natural number concepts. Trends Cogn Sci.

[131] Halberda J (2006). Multiple spatially overlapping sets can be enumerated in parallel. Psychol Sci.

[132] Lee K-M, Kang S-Y (2002). Arithmetic operation and working memory: differential suppression in dual tasks. Cognition.

[133] Michaux N (2013). Selective interference of finger movements on basic addition and subtraction problem solving. Exp Psychol.

[134] Mix KS (2016). Separate but correlated: the latent structure of space and mathematics across development. J Exp Psychol Gen.

